# Citrulline Malate Does Not Improve Muscle Recovery after Resistance Exercise in Untrained Young Adult Men

**DOI:** 10.3390/nu9101132

**Published:** 2017-10-18

**Authors:** Douglas K. da Silva, Jeferson L. Jacinto, Walquiria B. de Andrade, Mirela C. Roveratti, José M. Estoche, Mario C. W. Balvedi, Douglas B. de Oliveira, Rubens A. da Silva, Andreo F. Aguiar

**Affiliations:** 1 Center for Research in Health Sciences, North University of Paraná (UNOPAR), 675 Paris Ave., Londrina 86041-120, Brazil; douglaskratki@gmail.com (D.K.d.S.); jeferson1995lucas@gmail.com (J.L.J.); walquiria.fel@gmail.com (W.B.d.A.); fitmirela@gmail.com (M.C.R.); zezinhopersonal@outlook.com (J.M.E.); mariobalvedi@hotmail.com (M.C.W.B.); douglas_oliveira92@hotmail.com (D.B.d.O.); 2 Laboratory of Functional Evaluation and Human Motor Performance, LAFUP, Center for Research in Health Sciences, North Univeristy of Paraná (UNOPAR), 675 Paris Ave., Londrina 86041-120, Brazil; rubensalex@hotmail.com; 3 Département des Sciences de la Santé, Programme de Physiothérapie de L’université McGill Offert en Extension à L’UNIVERSITÉ du Québec à Chicoutimi (UQAC), 555 boul. De L’université, ville du Saguenay, Québec, QC G7H 5B8, Canada

**Keywords:** supplementation, skeletal muscle, weight training, amino acids, protein, exercise

## Abstract

The effects of citrulline malate (CM) on muscle recovery from resistance exercise remains unknown. We aimed to determine if citrulline malate supplementation improves muscle recovery after a single session of high-intensity resistance exercise (RE) in untrained young adult men. Nine young adult men (24.0 ± 3.3 years) participated in a double-blind crossover study in which they received 6 g of CM and placebo (PL) on two occasions, separated by a seven-day washout period. Each occasion consisted of a single session of high-intensity RE (0 h) and three subsequent fatigue tests sessions (at 24, 48, and 72 h) to assess the time course of muscle recovery. During the tests sessions, we assessed the following variables: number of maximum repetitions, electromyographic signal (i.e., root mean square (RMS) and median frequency (MF)), muscle soreness and perceived exertion, as well as blood levels of creatine kinase (CK), lactate, insulin, and testosterone:cortisol ratio. CK levels increased at 24 h post-exercise and remained elevate at 48 and 72 h, with no difference between CM and PL conditions. Muscle soreness increased at 24 h post-exercise, which progressively returned to baseline at 72 h in both conditions. Lactate levels increased immediately post-exercise and remained elevated at 24, 48, and 72 h in both conditions. No significant treatment × time interaction was found for all dependents variables (maximum repetitions, perceived exertion, CK, lactate, RMS, MF, and testosterone:cortisol ratio) during the recovery period. In conclusion, our data indicate that CM supplementation (single 6 g dose pre-workout) does not improve the muscle recovery process following a high-intensity RE session in untrained young adult men.

## 1. Introduction

Nutritional supplementation is a common strategy used by athletes and recreationally active adults to improve physical performance and muscle recovery. Nitric oxide (NO)-related supplements have received special attention for their possible ergogenic effects [1,2,3]. In particular, citrulline malate (CM) has been reported to augment aerobic energy production during exercise and increase phosphocreatine (PCr) during exercise recovery [2], improve ammonia (NH3) elimination during recovery from exhaustive exercise [4,5], attenuate muscle soreness after high-intensity resistance exercise (RE) [6], and increase performance during repeated bouts of high-intensity RE [6,7,8]. 

CM is formed by combination of L-citrulline (CIT) and malate (or malic acid)—a salt primarily found in apples. The potential ergogenic effects of CM have been attributed to three key mechanisms. First, oral CIT ingestion has been show to increase plasma L-arginine levels [9,10,11] at rest and exercise in humans. Given that L-arginine is the main substrate for synthesis of NO, an important modulator of blood flow [12], it has been suggested that oral CM supplementation may indirectly increase NO synthesis [6] and thus increase blood flow to active muscles. In this way, CM supplementation could contribute to increase nutrient delivery and/or clearance of waste products [13,14] such as plasma lactate and ammonia, thereby improving muscle function [15]. 

Second, CIT is an essential component of the urea cycle in the liver [16], where L-arginine produced from CIT is catabolized by arginase into ornithine and urea. Given that urea is the major vehicle to eliminate ammonia, a promoter of muscle fatigue via anaerobic glycolysis and consequent lactic acid production [17], it has been suggested that CIT supplementation may improve ammonia homeostasis [18] and thus improving muscle function. Third, malate is an intermediate of the tricarboxylic acid (TCA) cycle, and its greater availability after CM supplementation may augment aerobic ATP production by the TCA cycle through anaplerotic reactions [2], resulting in decreased muscle fatigue and improved muscle performance [2,6,7]. 

Based on the aforementioned mechanisms, it is postulated that CM supplementation may increase muscle performance and recovery by several ways, including reducing muscle soreness [6] and fatigue [2], improving oxygen delivery to the muscle [2], increasing oxidative ATP production during exercise and increasing PCr during exercise recovery [2], and lowering lactate and ammonium production [19]. Despite an abundance of studies on ergogenic effects of CM supplementation, no study to date has examined whether CM supplementation improves recovery of muscle function after high-intensity RE in young adult subjects. 

The aim of this study was to investigate the effects of free CM supplementation on recovery of muscle function after a single session of high-intensity RE in untrained young adult subjects. Based on the physiological properties and beneficial effects of CM on muscle fatigue and performance [6,7,8], we hypothesized that free CM supplementation would enhance muscle recovery from RE by improving muscle functional, metabolic, anabolic, and physiological responses.

## 2. Materials and Methods

### 2.1. Subjects 

Twelve recreationally active males were recruited from a university population, and 9 of them completed the study (three withdrew due to factors not related to the study). An a priori power analysis was conducted for an F test (repeated measures, within-between interaction factors for three-time points) to assess the required number of participants. We used the software G-Power^®^ (version 3.1; Dusseldorf, Germany) for the calculation. On the basis of a statistical power (1−β) of 0.80, a moderate effect size (0.55), and an overall level of significance of 0.05, at least eight subjects were required for this study. Participants were excluded if they: (1) were vegetarian or smoker; (2) had ingested any ergogenic supplement or anabolic steroids for the six months prior to the start of study; (3) were taking any medication that could affect muscle recovery or the ability to train intensely; (4) had participated in a exercise training program (more than two days per week, for six months prior to the beginning of the study); (5) were unable to provide a detailed description of their lifestyle and daily food intake; and (6) did not have medical approval to perform physical exercise. The participants’ characteristics are presented in Table 1. Four participants were overweight (mean BMI: 27.9 ± 1.33 kg/m^2^). All subjects were informed of the procedures, risks, and benefits of the investigation and signed an informed consent document approved by the Institutional Review Board of the University (protocol # 1.748.002). All procedures were carried out following the principles of the Declaration of Helsinki of 1975, revised in 2008.

### 2.2. Experimental Design 

**Figure 1:**
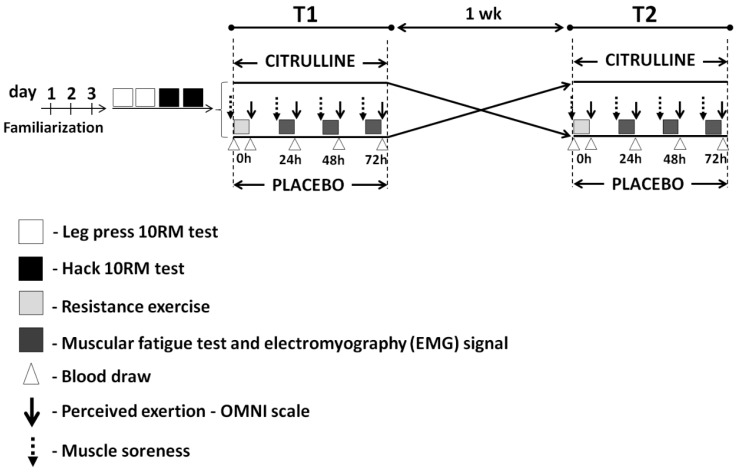
Experimental design.

A double-blind randomized crossover design was performed to examine the effects of CM supplementation on the time course of muscle recovery after a single session of high-intensity RE in young adult men (Figure 1). All subjects were randomly supplemented with 1 of 2 treatments (citrulline malate (CM) or placebo (PL)) on 2 occasions (T1 and T2) separated by a seven-day washout period in order to ensure complete CIT elimination from the body. Before T1, all subjects completed three sessions of familiarization and two sessions of 10-repetition maximum (10RM) tests for leg press and hack squat exercises. During T1 and T2, all subjects underwent a single session of RE (0 h) and 3 subsequent sessions (at 24, 48, and 72 h) of fatigue tests (to assess the time course of muscle recovery), performed at 60 min after supplementation. In each session of the fatigue test, functional (i.e., number of maximum repetitions, muscle soreness, and perceived exertion), metabolic (i.e., creatine quinase (CK) and lactate), anabolic (i.e., testosterone:cortisol ratio), and physiological (i.e., RMS and MF signal) indicators of muscle recovery were assessed. All participants completed a three-day dietary intake record (including one weekend day) during each study moment. All subjects had adequate protein (>1.2 g/kg/day) and CHO (>3.0 g/kg/day) intake during the study period (Table 2), according to the recommendations proposed by the American College of Sports Medicine (ACSM) [20].

### 2.3. Familiarization Protocol

Before T1, all subjects completed three familiarization sessions with leg press and hack squat exercises in order to minimize any potential learning effects and establish the reliability of the testing protocols (Figure 1). The protocol consisted of three sets of 8–12 repetitions with 2 min rest between sets and exercises. Maximal effort was requested in each exercise during the last two sessions in an attempt to achieve the approximate load for 10 repetitions maximum. Qualified personnel individually supervised each participant during the familiarization period. All session of familiarization were performed at the same location, between 8:00 and 10:00 a.m.

### 2.4. Determination of 10 Repetition Maximum Load

After the familiarization, the participants completed two sessions of 10 RM tests for leg press and hack squat exercises (Figure 1). The 10 RM test was preceded by a set of warm-up exercise (~15 repetitions) for each exercise. After 2 min of rest, the 10 RM attempts were performed with a progressively increasing load (5–10%) for each attempt, and the attempts were separated by 4 to 5 min rest intervals to allow adequate recovery. Only three attempts were allowed in each testing session. Verbal encouragement was provided during all 10 RM attempts. The exercises were standardized and continuously monitored by the same experienced rater in an attempt to assure the data quality and determine the 10 RM within three attempts. The intraclass correlation coefficients (ICC) test-retest were ≥0.93 for each 10 RM test, indicating the elimination of the learning curve for the subjects. All session of 10 RM tests were performed at the same location, between 8:00 and 10:00 am.

### 2.5. Resistance Exercise 

During T1 and T2, all subjects were submitted to a single session of high-intensity RE (3 sets of 8–12 repetitions at 90% of 10 RM with 2 min rest between sets and exercises), involving the leg press and hack squat exercises (bilateral), using commercial machines (Nakagym equipment, São Paulo, Brazil). The cadence of muscle action was 1 s concentric: 2 s eccentric according metronome. This protocol was designed to maximize the recruitment of quadriceps muscle, and the training stimulus was similar to a conventional RE session (3 sets of 8–12 repetitions at 70–75% of 1RM) for novice individuals [21]. We have chosen to use the 10RM load (as opposed to 1RM) due to its easy application in the practical context and safety for novice practitioners. RE session began with general (moderate walking on treadmill for 10 min) and specific (1 set of 12 repetitions with a self-selected load) warm-up exercises for quadriceps muscle. Qualified personnel supervised each participant individually during every workout. The RT sessions were performed at the same location, between 8:00 and 10:00 a.m.

### 2.6. Supplementation Protocol

During T1 and T2, the subjects orally ingested an identical looking and equivalent amount (6 g) of citrulline malate (CM) or placebo-cornstarch (PL) dissolved in 200 mL of water. CM and PL were analyzed and confirmed for purity prior to the study. We chose to use a dose of 6 g based on prior studies that showed an increase in plasma citrulline (CIT) concentrations after intense exercise in cyclists without any adverse effects reported [10,22]. The supplementation was performed 60 min before the exercise sessions and fatigue tests, based on prior studies that reported an ergogenic effect of CM during multiple bouts of upper-body RE in young men [6,7]. To ensure the double-blind design, an individual who was not involved in the study was responsible for placing the supplements into bags and labeling the capsules with the subjects’ names according to the randomization list. 

### 2.7. Dietary Intake 

Participant completed a three-day dietary intake record (including one weekend day) during the T1 and T2 moments. The macronutrient composition of the diets was calculated using software for nutritional assessment (Avanutri, version 3.1.4, Rio de Janeiro-RJ, Brazil). Participants were instructed to maintain their habitual daily diet, and to refrain from any strenuous activity during experimental period. The participants were also instructed to report any adverse events from the supplements. No discomfort or adverse effects were reported after CM ingestion.

### 2.8. Perceived Exertion

Rating of perceived exertion was measured immediately after muscular fatigue tests (24, 48, and 72 h after RE session) during T1 and T2 using the OMNI-RES scale [23] (Figure 1). The subjects were instructed to report the perceived exertion value indicating a number of the OMNI-RES scale (0 “no effort” and 10 “maximal effort”) that best represented their overall muscular effort [23,24]. The score was the value (0–10) reported in OMNI-RES scale.

### 2.9. Delayed-Onset Muscle Soreness

Muscle soreness was measured before muscular endurance tests (24, 48, and 72 h after RE session) during T1 and T2 using a visual analog scale (VAS) (Figure 1). The VAS consists of a 10-cm line whose end points were labeled with “no pain” (left) and “unbearable pain” (right). The subjects were instructed to palpate their quadriceps muscle and mark a vertical line at the scale that best represented their rating of momentary soreness. The score was the distance (in cm) from the left side of the scale to the point marked [25].

### 2.10. Muscular Endurance Tests and Electromyographic (EMG) Signal Recordings

During T1 and T2, all subjects were submitted to a muscular fatigue test (one set at 100% of 10 RM until failure) in the leg press and hack squat exercises at 24, 48, and 72 h after RT session (Figure 1) in order to examine the muscular endurance recovery. During the leg press test, surface EMG signals were recorded from the vastus lateralis (VL) muscle using a pre-amplified (gain: 1000) active bipolar surface electrode (Model EMG System Brazil Ltda, São José dos Campos, São Paulo, Brazil) at a sampling rate of 2000 Hz. The subjects’ skin was prepared by removing the superficial dead skin and was sterilized with an alcohol swab. The electrode was placed on a location near the center of the belly of the muscle according to the SENIAM (Surface EMG for Non-Invasive Assessment of Muscles) recommendations and from our previous work [26], and the reference electrode was fixed at the right styloid process. The EMG signals were filtered with a band-pass digital filter between 10 and 500 Hz to remove high frequency noise as well as low-frequency movement.

To determine the muscle activation and fatigue, two EMG parameters were computed: root mean square (RMS) and median frequency (MF). RMS corresponds to the muscular activation of the second and before last contractions of the endurance test (e.g., to avoid the acceleration and the deceleration portions of the concentric leg contractions during the extension phase of movement). This parameter was computed by a moving RMS method executed on successive 250 ms (512 points) time-series windows (50% overlap) to obtain the RMS average values during the entire leg press and hack squat exercises. MF corresponds to muscle fatigue from the magnitude of the electromyographic spectral content evaluated by the MF value of the power spectra (Short-fast Fourier transform, Hanning window processing) during successive time windows (50% overlap) of 250 ms for the total time of the fatigue test. Least squares linear regression analysis was applied to the MF time series to calculate the rate of decline in MF over time (MF/time slope as a muscle fatigue index), as supported by previous studies [27,28,29]. All EMG signals and estimates (RMS and MF) were processed using MATLAB sub-routines for data computation from subsequent analysis (Version 8.0, Mathworks^®^, South Natick, MA, USA).

### 2.11. Blood Collection and Analysis

Blood samples were collected at pre- and post-exercise as well as immediately after muscular fatigue tests (24, 48, and 72 h after RE session) during T1 and T2 for analyses of CK, lactate, insulin, glucose, cholesterol, testosterone, and cortisol concentrations (Figure 1). The blood samples were allowed to coagulate at room temperature for 60 min and then centrifuged at 2000× *g* for 15 min, and the serum was frozen at −80 °C until analysis. All analyses were performed in a laboratory equipped with automated systems using commercial kits for chemiluminescence (insulin, testosterone, and cortisol), kinetic (CK and lactate), and enzymatic (cholesterol, glucose) techniques. 

### 2.12. Statistical Analyses

Data are expressed as means ± standard deviation (SD). The normality and homogeneity for outcome measures were tested using the Shapiro-Wilk’s and Levene’s tests, respectively. Independent variables included the supplementation protocol (i.e., CM and PL) and time (i.e., 24, 48, and 72 h). Dependent variables included number of maximum repetitions, perceived exertion, muscle soreness, testosterone:cortisol ratio, and EMG signs (i.e., RMS and MF). Food intake (during T1 and T2) and area under curve (AUC) were analyzed using a paired *t*-test. Two-way (treatment × time) analysis of variance (ANOVA) with repeated measures was used to evaluate changes over time and between groups for all dependent variables. Violation of sphericity was adjusted by Greenhouse-Geisser. When significant differences were confirmed with ANOVA, multiple comparisons testing were performed using Bonferroni post hoc correction to identify these differences. The significance level was set at *p* ≤ 0.05. Statistical analyses were performed using SPSS statistical analysis software (SPSS version 20.0; Chicago, IL, USA).

Effect size (ES) and confidential interval (CI) have been recommended as a more appropriate analysis for evidence the magnitude of an intervention (treatment effect) [30,31] due their biological importance to make clinical decisions [31], and elimination of confounding factors such as sample size and measure variability [32,33,34]. Thus, we also analyzed ES and 95% CI (see supplemental material in Figure S1) [35] to ensure a more realistic biological interpretation of data, and to provide practical information on the magnitude or direction of the difference (treatment effect).

## 3. Results

### 3.1. Dietary Intake

The macronutrient intake is presented in Table 2. No significant difference in the daily dietary intake were observed between T1 and T2 moments, and all participants had adequate protein (>1.2 g/kg/day) and CHO (>3 g/kg/day) intake during the study period, according to the recommendations proposed by the American College of Sports Medicine (ACSM) [20]. No adverse effect was reported by participant throughout study.

### 3.2. Total Repetitions and Perceived Exertion in the Fatigue Tests during Recovery

**Figure 2:**
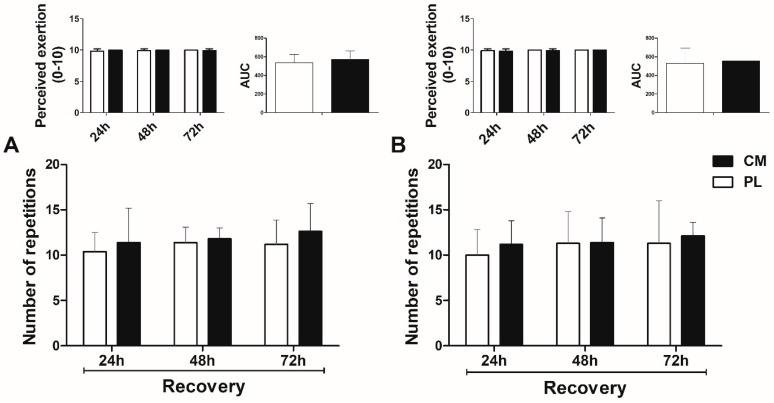
Number of maximum repetitions in the leg press (**A**) and hack squat (**B**) fatigue tests during recovery period at 24, 48, and 72 h post-exercise. Citrulline malate *CM*, Placebo *PL*. Left upper figure indicates the perceived exertion—OMNI scale (0–10) immediately after each test. Right upper figure indicates the area under curve (AUC) for 3-d recovery period. (repeated-measures ANOVA: time *p* > 0.05; Treatment x time *p* > 0.05). Data are means ± SD.

The total number of repetitions and perceived exertion for leg press (Figure 2A) and hack squat (Figure 2B) fatigue tests during recovery at 24, 48, and 72 h post-exercise did not differ over time between CM and PL conditions (treatment × time, leg press *p* = 0.703, hack squat *p* = 0.656; time, leg press *p* = 0.287, hack squat *p* = 0.186). Both CM and PL conditions achieved maximum perceived effort (OMNI scale score) in leg press (CM: 10 ± 0 vs. PL: 10 ± 0) and hack squat (CM: 10 ± 0 vs. PL: 10 ± 0) fatigue tests (Figure 2A,B, respectively), with no significant treatment × time interaction (leg press *p* = 0.104, hack squat *p* = 0.709) and main time effect (leg press *p* = 0.709, hack squat *p* = 0.104). The ES and 95% CI for muscle recovery variables (Total repetitions, CK, lactate, RMS and soreness) at 24, 48, and 72 h post-exercise are shown in Supplemental Figure S1. The data show no positive effect in favor of CM for all variables. 

### 3.3. CK and Lactate Blood Levels after Fatigue Test during Recovery

**Figure 3:**
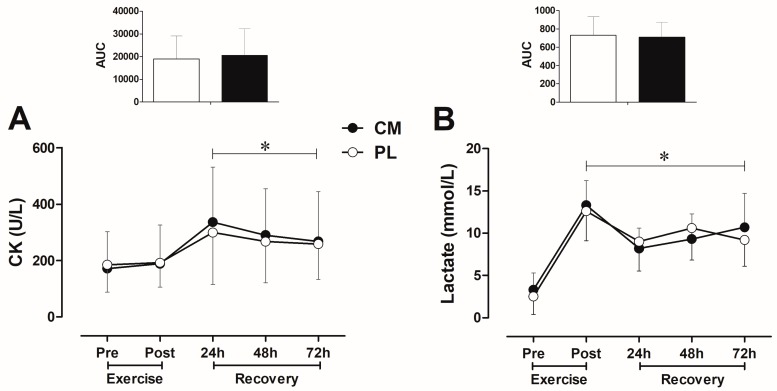
Blood CK (**A**) and lactate (**B**) levels at pre- and post-exercise session, and immediately after fatigue tests during recovery (at 24, 48, and 72 h post-exercise). Citrulline malate (CM), Placebo (PL). Upper figure indicates the area under curve (AUC) from pre- to 72 h post-exercise. (Repeated-measures ANOVA: * *p* < 0.05 compared to pretraining). Data are means ± SD.

Serum CK levels increased (time *p* < 0.001) at 24 h post-exercise and remained elevated at 48 and 72 h (Figure 3A), with no differences between CM and PL conditions (treatment × time *p* = 0.632) (Figure 3A). Plasma lactate levels increased (time *p* < 0.001) immediately post-exercise (Figure 3B) and remained elevated at 24, 48, and 72 h in both conditions (treatment × time *p* = 0.456). 

### 3.4. Muscle Soreness during Recovery

Perceived intensity of muscle soreness decreased (time *p* = 0.004) from 24 h (CM: 4.2 ± 3.3 and PL: 2.9 ± 1.8 cm) and 48 h (CM: 4.1 ± 3.8 and PL: 3.2 ± 2.5 cm) to 72 h post-exercise (CM: 2.3 ± 2.0 and PL: 1.6 ± 2.0 cm), with no differences between CM and PL conditions (treatment × time *p* = 0.819). 

### 3.5. Testosterone: Cortisol Ratio after Fatigue Test during Recovery 

**Figure 4:**
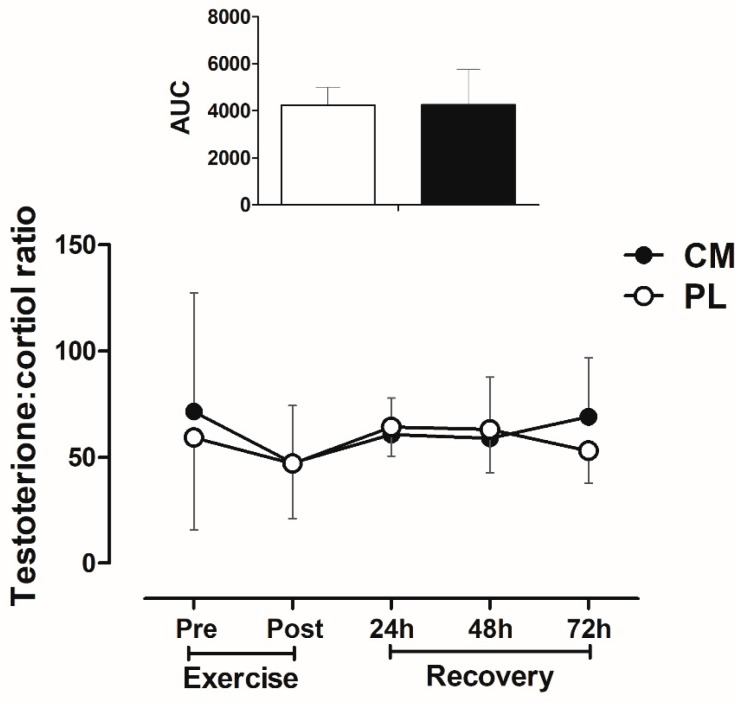
Serum testosterone:cortisol ratio at pre- and post-exercise, and immediately after fatigue tests during recovery (at 24, 48, and 72 h post-exercise). Citrulline malate (CM), Placebo (PL). Upper figure indicates the area under curve (AUC) from pre- to 72 h post-exercise. (Repeated-measures ANOVA: time *p* > 0.05; Treatment x time *p* > 0.05). Data are means ± SD.

Serum testosterone: cortisol ratio (Figure 4) did not differ between CM and PL conditions during recovery period at 24, 48, and 72 h post-exercise (treatment × time *p* = 0.970; time *p* = 0.947). 

### 3.6. EMG Signal in the Fatigue Test during Recovery

**Figure 5:**
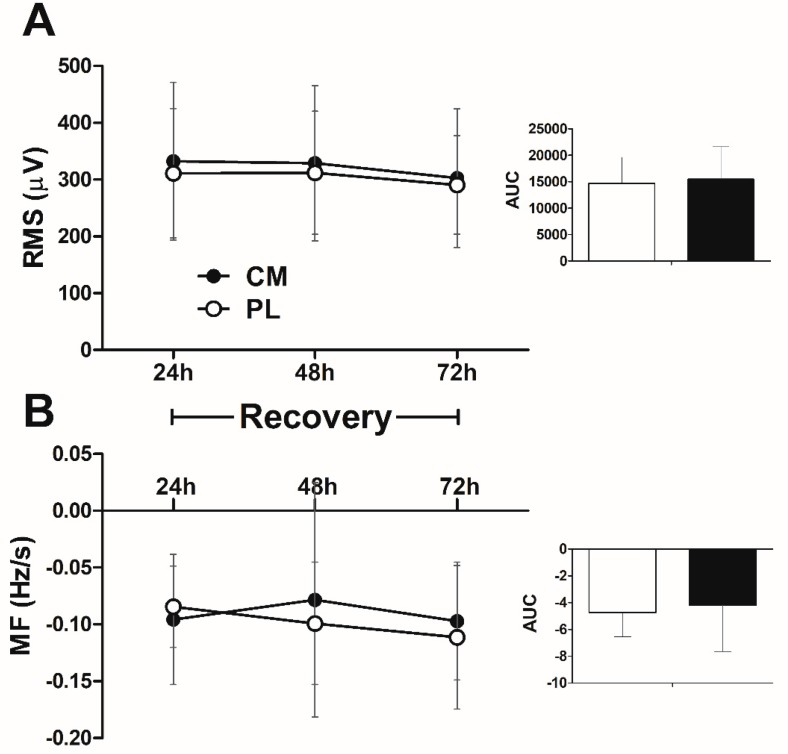
RMS (**A**) and MF (**B**) values in the leg press fatigue test at 24, 48, and 72 h after exercise session. Citrulline malate (CM), Placebo (PL). Right figure indicates the area under curve (AUC) for the three-day recovery period. (Repeated-measures ANOVA: time *p* > 0.05; Treatment x time *p* > 0.05). Data are means ± SD.

RMS (Figure 5A) and MF slope (Figure 5B) in the leg press fatigue test during the recovery period (24, 48, and 72 h after RT) did not change over time between CM and PL conditions (treatment × time, RMS *p* = 0.937, MF slope *p* = 0.493; time, RMS *p* = 0.111, MF slope *p* = 0.483). Total RMS (CM: 321 ± 128 µV vs. PL: 304 ± 100 µV; *p* = 0.217) and MF slope (CM: −0.090 ± 0.073 Hz/s vs. PL: −0.098 ± 0.051 Hz/s; *p* = 0.586) over the three times of recovery were similar between CM and PL conditions.

## 4. Discussion

To our knowledge, this is the first study to examine the effects of free CM supplementation on the time course of muscle recovery after a single session of high-intensity RE in untrained young adult men. Based on the physiological properties and beneficial effects of CM on fatigue and performance [2,6,7,8], we hypothesized that CM supplementation would enhance muscle recovery from RE by improving the muscular functional, metabolic, anabolic, and physiological responses. In contrast to the hypothesis, we observed that free CM supplementation (6 g at 60 min pre-workout) does not improve the major functional (i.e., number of maximum repetitions, muscle soreness, and perceived exertion), metabolic (i.e., CK, and lactate), anabolic (i.e., testosterone:cortisol ratio), and physiological (i.e., RMS and MF signal) indicators of muscle recovery in untrained young adult men.

Given that L-arginine is the main substrate for the NO synthesis, and CIT may be converted to L-arginine in kidney [36], it has been postulated that oral CM supplementation may increase NO synthesis [6] and, consequently, improve muscle blood flow for active muscles. As a result, CM supplementation could increase nutrient and oxygen delivery to the muscle and/or enhance clearance of waste-products [13,14] such as plasma lactate and ammonia, thereby improving muscle function/recovery from exercise [15]. Nevertheless, we have shown that CM supplementation does not promote any improvements in markers of muscle function (i.e., number of maximum repetitions, muscle soreness, and perceived exertion) during recovery from RE (24, 48, ad 72 h). In contrast to our results, previous performance studies have shown that CM supplementation (8 g) can reduce rating of perceived exertion [8] and increase performance (i.e., number of maximum repetitions) during repeated bouts of high-intensity RE to failure [6,7,8]. Although a direct comparison of our findings with the aforementioned studies may not be warranted due to the differences between the studies aims (e.g., recovery vs. performance), an important question is why we did not see improved muscle function during recovery, as others have reported in performance studies [6,7,8].

A possible explanation may be the inability of CM to attenuate muscle damage during recovery period. The RE session resulted in similar increase in plasma CK (24 h post-exercise) and lactate (immediately post-exercise) levels in both the CM and PL conditions, which typically indicates muscle damage and impaired muscle function. Wax et al. [7] reported an increase in muscle performance (i.e., number of repetitions performed) without any inhibitory effect of CM on lactate production, indicating that beneficial effects of CM on muscle function are not attributed to lactate reduction. Similarly, Vanuxem et al. [4] showed no difference in lactate levels between CM and PL groups during a maximal exercise test. Therefore, it seems likely that the lack of effects of CM on muscle function (i.e., number of maximum repetition) observed in our study is more related to inability of CM to attenuate RE-induced muscle damage (indicate by similar CK levels between CM and PL during recovery period) than to reduce lactate production. Considering that muscle damage is a potential indicator of impaired muscle function after intense exercise [37], its negative impact could be superior to any small beneficial effects of CM supplementation on muscle function during recovery. This could explain, at least partially, why CM supplementation promoted beneficial effects when muscle was tested in normal conditions (without any exercise-induced damage) [6,7,8], but not in our study, where the muscle was exposed to a high degree of muscle damage after RE session. 

The absence of a positive effect of CM on muscle regeneration and function during recovery may be due to its particular mechanisms of action. CM has been shown to increase ATP production and phosphocreatine (PCr) resynthesis rate [2] and improve ammonia (NH_3_) elimination during recovery from exhaustive exercise [4,5], but CIT does not directly increase muscle protein synthesis rates or the phosphorylation of anabolic signaling proteins (i.e., mammalian target of rapamycin (mTOR)) in human muscles [38]. This lack of CIT effects on muscular regeneration markers [38] was supported by the absence of differences in anabolic hormones (i.e., testosterone:cortisol ratio) levels between CM and PL conditions in our study. Therefore, it is likely that the inability to improve anabolic factors result in no beneficial effect of CM supplementation on muscle regeneration (i.e., CK levels) and function (i.e., number of maximum repetition) during recovery from RE. This was also evidenced by the observation that muscle soreness did not improve with CM supplementation. 

Additionally, we showed no differences in electromyographic (EMG) indicators of muscle activation (RMS) and fatigue (MF) between CM and PL conditions. To our knowledge, this is the first study to examine the effects of CM supplementation on EMG indicators of muscle activation (RMS) and fatigue (MF) during recovery from high-intensity RE. Considering that the decline in MF and increase in RMS are typically associated with muscle fatigue during isometric and dynamic contractions [39,40,41,42], we expected an inverse effect of CIT supplementation on these factors in the fatigue tests during recovery period. However, no differences were observed between the CM and PL conditions, which is in line with the lack of a beneficial effect of CM supplementation on muscle function (i.e., number of repetitions performed). This indicates that CM supplementation is not effective in improving the neuromuscular responses during recovery from RE. This result is consistent with no difference in the muscle soreness between CM and PL conditions during the recovery period. Although muscle soreness may be a poor indicator of exercise-induced muscle damage during recovery [43], it usually reflects muscle fatigue. This supports the findings of the present study that CM supplementation does not attenuate muscle fatigue during the course of recovery. Therefore, any possible beneficial effects of CM supplementation on muscle soreness [6], fatigue [2], oxygen delivery to the muscle [2], aerobic energy production [2], and lactate and ammonium clearance [18] may not be sufficient to improve muscle recovery in untrained young adult men. It is noteworthy that a dose of 6 g of CM is sufficient to increase plasma CIT (~173% increase) and arginine (~123% increase) levels [10], and NO production after exercise (measured as nitrite levels) [10,22]. In addition, is has been demonstrated that after administration of CIT (5–10 g), time to reach maximum concentration (Tmax) was ~43 min and decreased to baseline by 3–5 h [44]. Thus, the lack of a positive effect of CM supplementation on muscle recovery from RE does not necessarily indicate that our subjects did not experience an increase in the plasma CIT and arginine levels during and after exercise.

A few limitations of this study must be mentioned. First, we did not analyze plasma NO and CIT concentrations. However, previous studies that used the same dose of CIT (i.e., 6 g) showed an increase in plasma CIT concentrations [10,22]. Second, we did not collect muscle biopsies for analysis of muscle tissue markers of regeneration (e.g., protein synthesis and anabolic factors such as insulin-like growth factor (IGF-I), hepatocyte growth factor (HGF), mTOR, and ribosomal protein S6 kinase (p70S6k)) and damage (e.g., histological changes); however, we analyzed the major plasma markers of muscle damage and regeneration (i.e., CK and testosterone:cortisol ratio) as well as functional outcome measures (i.e., number of repetitions performed, perceived exertion, muscle soreness, and EMG signs). Finally, we did not standardize the subjects’ diets. However, we did instruct subjects to duplicate their food intake for the 24 h proceeding each session. 

In conclusion, CM supplementation (6 g dose at 60 min pre-workout) does not improve muscle recovery from a single session of high-intensity RE in untrained young adult men. Therefore, it is premature to recommend free CM supplementation as an ergogenic aid to improve muscle recovery after RE. Future studies are required to assess the effects of CM in other populations (e.g., elderly and/or women) with different training status (e.g., recreational practitioners and/or athletes). 

## Figures and Tables

**Table 1:** Participant characteristics.

**Age, year**	24.0 ± 3.3
**Height, cm**	175.1 ± 4.8
**Weight, kg**	77.4 ± 9.6
**BMI, kg/m^2^**	25.3 ± 2.9
**Insulin, µIU/mL**	9.3 ± 6.4
**Glucose, mg/dL**	71.9 ± 19.0
**Cholesterol, mg/dL**	155.2 ± 31.0

Values are means ± standard deviation (SD).

**Table 2:** Macronutrient dietary intake between T1 and T2 moments.

	T1	T2	*p* Value
Protein, g/kg/day	1.8 ± 1.0	1.7 ± 0.8	0.51
Carbohydrate, g/kg/day	4.6 ± 1.6	4.9 ± 1.4	0.31
Fat, g/kg/day	1.1 ± 0.6	1.2 ± 0.8	0.67
Total energy, kcal	2681 ± 806	2874 ± 1058	0.47
Total energy, kJ	11,218 ± 3373	12,027 ± 4429	0.47

Values are means ± standard deviation (SD). There were no differences between T1 and T2 moments.

## Supplementary Materials

The following are available online at www.mdpi.com/2072-6643/9/10/1132/s1, Figure S1: Effect size (95% CI) for muscle recovery variables (Number of maximum repetitions in the leg press (A) and hack squat (B), levels of CK (C), lactate (D), and values of RMS (E) and soreness (F)) between CM and PL at 24, 48, and 72 h post-exercise.
