# Supplementary figures and images for: Citrulline Malate Does Not Improve Muscle Recovery after Resistance Exercise in Untrained Young Adult Men
Source: Nutrients. 2017 Oct 18;9(10):1132. doi: 10.3390/nu9101132 (PMC5691748; doi:10.3390/nu9101132)

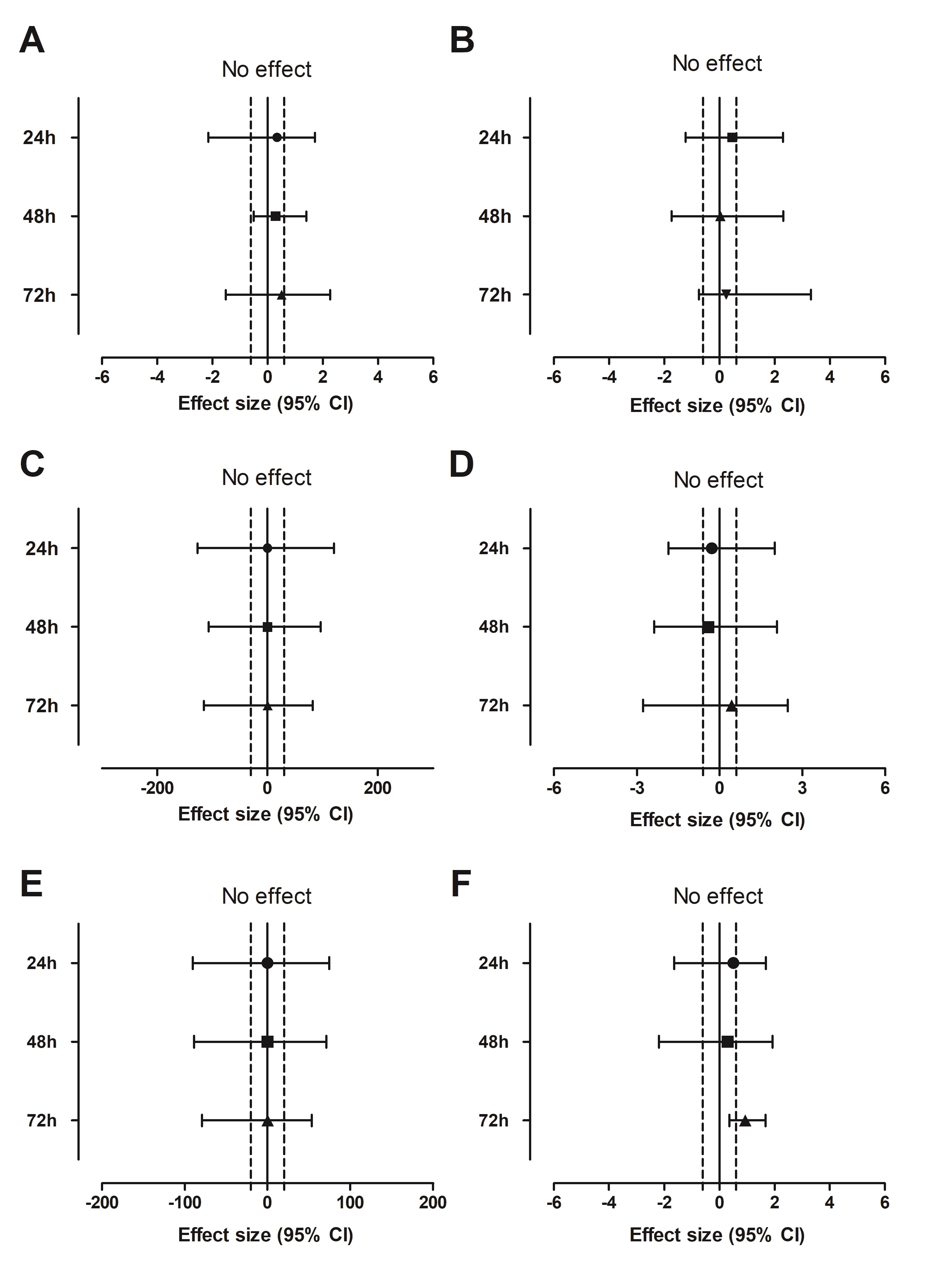

Supplement: Supplementary file 1 [file nutrients-09-01132-s001.jpg]
